# Dual-Drug Delivery via the Self-Assembled Conjugates of Choline-Functionalized Graft Copolymers

**DOI:** 10.3390/ma15134457

**Published:** 2022-06-24

**Authors:** Katarzyna Niesyto, Aleksy Mazur, Dorota Neugebauer

**Affiliations:** Department of Physical Chemistry and Technology of Polymers, Faculty of Chemistry, Silesian University of Technology, 44-100 Gliwice, Poland; katarzyna.niesyto@polsl.pl (K.N.); aleksy.mazur@gmail.com (A.M.)

**Keywords:** graft copolymers, dual-drug delivery systems, polymer carriers

## Abstract

Graft copolymers based on a choline ionic liquid (IL), [2-(methacryloyloxy)ethyl]-trimethylammonium chloride (TMAMA), were obtained by atom transfer radical polymerization. The presence of chloride counterions in the trimethylammonium groups promoted anion exchange to introduce fusidate anions (FUS, 32–55 mol.%) as the pharmaceutical anions. Both the choline-based IL copolymers and their ionic drug-carrier conjugates (FUS systems as the first type, 26–208 nm) formed micellar structures (CMC = 0.011–0.025 mg/mL). The amphiphilic systems were advantageous for the encapsulation of rifampicin (RIF, 40–67 mol.%), a well-known antibiotic, resulting in single-drug (RIF systems as the second type, 40–95 nm) and dual-drug systems (FUS/RIF as the third type, 31–65 nm). The obtained systems released significant amounts of drugs (FUS > RIF), which could be adjusted by the content of ionic units and the length of the copolymer side chains. The dual-drug systems released 31–55% FUS (4.3–5.6 μg/mL) and 19–31% RIF (3.3–4.0 μg/mL), and these results were slightly lower than those for the single-drug systems, reaching 45–81% for FUS (3.8–8.2 μg/mL) and 20–37% for RIF (3.4–4.0 μg/mL). The designed polymer systems show potential as co-delivery systems for combined therapy against drug-resistant strains using two drugs in one formula instead of the separate delivery of two drugs.

## 1. Introduction

Polymer nanocarriers have recently gained enormous attention due to their potential applications as fluorescent biosensors and markers [1,2], as well as in drug delivery systems (DDSs) [3,4,5,6]. A wide range of biocompatible polymer matrices can be pre-designed, and their properties can be adjusted to meet the specific needs of a given carrier [7]. Controlled drug release, with a limited increase in drug concentration in the body, is one of the main advantages of nanocarriers [8,9,10]. Generally, DDSs are used to improve the effectiveness of standard drugs and conventional treatments.

Depending on a polymer’s structure, drugs can be bound to a nanocarrier in various ways. Amphiphilic polymers are composed of hydrophobic and hydrophilic units, which allow them to form micellar structures, in which a drug can be physically encapsulated into the core [11,12]. Such structures can be obtained by coupling polymer segments or via the copolymerization of monomers with different solubilities. Of particular note are amphiphilic copolymers based on ionic liquids (ILs), which are considered to be green solvents. Due to their unique properties, such as chemical and thermal stability and high ionic conductivity, they have been applied in various industries [13,14]. The most important properties of DDSs are the high biocompatibility and low toxicity of many ILs, which can improve the pharmacokinetic and pharmacodynamic properties of the drugs being transported [15,16,17]. Some of them, i.e., cholinium-based ILs, show biological functions [18,19,20,21]. Micelles based on IL (co)polymers have been studied for the encapsulation and delivery of various active pharmaceutical ingredients (APIs), e.g., erythromycin, indomethacin, quercetin [22], curcumin [23,24], acyclovir [25], paclitaxel [26], dopamine [27], and doxorubicin [28,29].

The ionic structure of IL-based polymers can be applied for ionic exchange. This approach enables the introduction of APIs in ionic form into a polymer to produce ionic conjugates. Various cations, such as imidazolium, lidocainum, cholinium, and guanidinium, have been employed in polymers to carry ionic ibuprofenate [30,31], ampicillin [32,33], (acetyl)salicylate, or aspirin [34]. In the case of a matrix decorated with cholinium ILs, there are reports of anion exchange and the delivery of various anions based on mefenamic acid [35,36], nalidixic, niflumic, pyrazinoic, and picolinic acids [37], salicylate [22,38], *p*-aminosalicylate, clavulanate [39], fusidate, and piperacillin [40]. The aforementioned systems have focused on delivering a single drug introduced via encapsulation or ion exchange. The specificity of IL-based polymers that also exhibit amphiphilicity favors the combination of both of these abilities to obtain systems with a dual pharmacological action. In this case, the biological activity of the polyIL (PIL) conjugate conferred by the ionic drug can be doubled by encapsulating a second drug (non-ionic) into the micelle core. 

**Figure 1:**
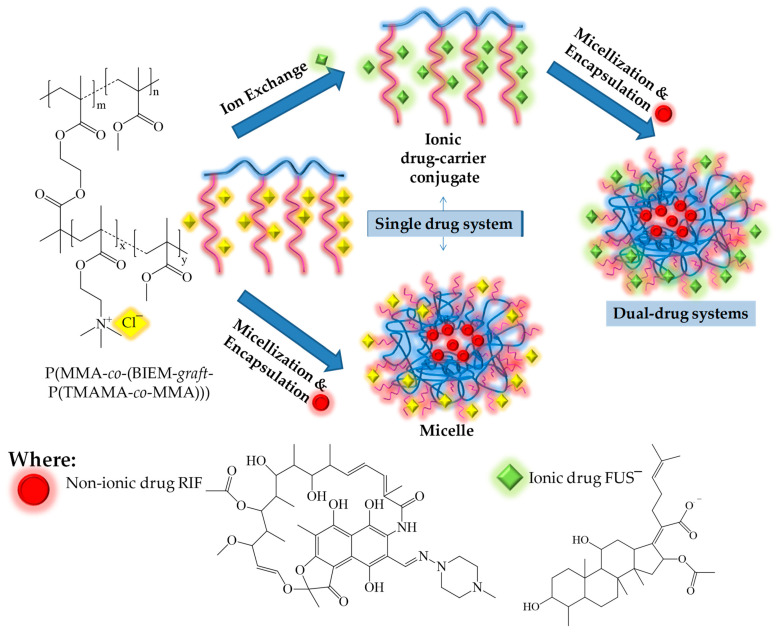
Schematic route of the amphiphilic graft copolymer based on TMAMA and various types of drug carriers.

Herein, we report PIL grafted copolymers as the matrices for innovative dual-drug delivery systems based on micelles of ionic conjugates (Figure 1). These systems are advantageous, especially for combined therapies. For this purpose, our previously designed graft copolymers based on a polymerizable IL, [2-(methacryloyloxy)ethyl]trimethylammonium chloride (TMAMA) [39], were applied. This IL-monomer was chosen due to the presence of a biologically active choline group, its advantageous non-toxicity, and its high biocompatibility. Cytotoxicity tests indicated the non-toxic action of IL-graft copolymers against normal BEAS-2B cell lines. Moreover, their selective biological action was observed versus normal and lung cancer cell lines [21]. Furthermore, the ionic structure of IL units was convenient for the ionic exchange reaction, which in this study, was used to introduce fusidate anions (FUS^−^) into the side chains of the copolymer. The selected API anion corresponds to fusidic acid, which is a natural bacteriostatic antibiotic with a steroidal structure that can inhibit the synthesis of bacterial proteins. This drug is also used in the treatment of lung diseases due to its effective action against strains of, e.g., *Staphylococcus aureus*, *Bordetella pertussis*, *Mycobacterium leprae*, and *Mycobacterium tuberculosis.* The amphiphilicity of polymer-FUS conjugates encouraged us to encapsulate the antibacterial drug, rifampicin (RIF), which is conventionally applied for the treatment of tuberculosis. Bacterial resistance has been frequently noticed during treatment with fusidic acid alone; hence, a combination therapy based on drugs containing fusidic acid (and its sodium salt) and rifampicin was used to obtain a better treatment effect. However, a formulation containing both rifampicin and fusidate is not commercially available, and they are currently used as separate medicines, i.e., Rifampicin TZF and Fucidin^®^. In our studies, the efficiencies of dual DDSs (RIF/FUS^−^) were compared with the single DDSs (micellar with RIF vs. ionic conjugate with FUS^−^) by monitoring the content of the drugs and their in vitro release under conditions approximate to human fluids (phosphate-buffered saline, PBS at pH = 7.4) to show the improved applicability in relation to their single-drug carrier analogs.

## 2. Materials and Methods

Methyl methacrylate (MMA, Alfa Aesar, Warsaw, Poland) and [2-(methacryloyl-oxy)ethyl]trimethylammonium chloride (TMAMA, 80% aq. solution, Sigma-Aldrich, Poznan, Poland) were dried using molecular sieves or under vacuum, respectively. Copper(I) chloride (CuCl, Fluka, 98%, Steinheim, Germany) was purged according to procedures described previously [39]. Methanol was obtained from Chempur (Piekary Śląskie, Poland). Rifampicin (RIF, 97%) and sodium fusidate (FUS, 98.8%) were purchased from Alfa Aesar (Warsaw, Poland) and used without prior purification. Phosphate-buffered saline (PBS), 2,2′-bipyridine (bpy), and tetrahydrofuran (THF) were obtained from Sigma-Aldrich (Poznań, Poland).

### 2.1. Synthesis of Ionic Graft Copolymers Bearing Cl^−^ or FUS^−^

The preparation of copolymers of methyl methacrylate and 2-(2-bromoisobutyryloxy)ethyl methacrylate (P(MMA-*co*-BIEM)) with different contents of bromoester active groups (25% or 50%), which were used as the multifunctional macroinitiators (MI) in a “grafting-from” strategy, have been described previously [39].

Comonomers TMAMA (1.80 g, 8.66 mmol), MMA (0.913 mL, 8.57 mmol), methanol (2 mL), THF (1 mL), MI with 25% of initiating bromoester groups (97.17 mg), and bpy (27.07 mg, 0.18 mmol) were placed into a Schlenk flask. Two freeze-pump-thaw cycles were carried out, and then the catalyst CuCl (12.91 mg, 0.08 mmol) was added to the mixture. The reaction was carried out at 40 °C for 2 h. The reaction was stopped by exposing the mixture to air. The polymer was precipitated in a chloroform–diethyl ether mixture and then dried under vacuum.

The obtained graft copolymer I (21.0 mg, including 0.06 mmol of TMAMA units) was dissolved in 1 mL of methanol. Next, the sodium salt of fusidic acid (FUS, 29.8 mg; 0.06 mmol) was inserted into the polymer solution. The ion-exchange reaction was performed for 48 h at room temperature. The conjugate I_FUS was obtained after drying under reduced pressure. 

### 2.2. Encapsulation and Micellization

The amphiphilic graft copolymer (20 mg) and RIF in a weight ratio of 1:1 were dissolved in methanol (2 mL). Then, deionized water was dropped into the mixture (4 mL, two-fold excess of water relative to the solvent) and stirred for 24 h. Next, the methanol was evaporated, and the aqueous fraction was collected and lyophilized to obtain a solid product. 

The same procedure was applied to form single-drug systems based on copolymers with chloride anions and dual-drug systems, in which the conjugates with FUS anions were mixed with the non-ionic RIF.

### 2.3. Drug Release Studies of Ionic and Non-Ionic Drugs

The obtained conjugate/micelle/dual-system (1.0 mg) was dissolved in 1 mL of PBS solution (pH = 7.4). Next, the mixture (1 mL) was transferred to a dialysis membrane bag (MWCO = 3.5 kDa), which was placed in a glass vial filled with 45 mL of PBS. Drug release experiments were performed, under stirring, at 37 °C. The samples (0.5 mL) were taken at appropriate time intervals and mixed with 0.5 mL of methanol. The samples prepared in this way were analyzed on a UV-Vis spectrophotometer, observing the absorption maximum at λ = 207 nm for FUS^−^ and 330 nm for RIF.

### 2.4. Characterization

^1^H NMR spectra were recorded using a UNITY/NOVA (Varian, Mulgrave, Victoria, Australia) spectrometer operating at 300 MHz. The measurements were performed by dissolving samples in deuterated dimethyl sulfoxide (DMSO) with tetramethylsilane (TMS) as an internal standard. Molecular weight and dispersity index (M_n_ and Ð) were estimated by size-exclusion chromatography (SEC). Measurements were performed on a chromatograph (Ultimate 3000 with differential refractometer RefractoMax 521 detector, Thermo Fisher Scientific, Waltham, MA, USA) in DMF containing 10 mM LiBr at 50 °C with a flow rate of 0.25 mL/min using a TSKgel Guard SuperMPHZ-H 6 µm pre-column (4.6 mm × 2 cm) and two TSKgel SuperMultiporeHZ-H 6 µm columns (4.6 mm × 15 cm), or in water with a flow rate of 0.35 mL/min using a TSKgel SuperAW3000 4 μm column (6.0 mm × 15 cm) and a TSKgel SuperAW-H Guard pre-column (4.6 mm × 35 mm). The calculations were based on poly(ethylene oxide) (PEO) standards (982–969,000 g/mol). The critical micelle concentration (CMC) was evaluated by measuring the interfacial tension (IFT) using the pendant drop method on a goniometer (OCA 15EC, DataPhysics, Filderstadt, Germany). For this purpose, a series of aqueous polymer solutions (0.0006–0.06 mg/mL) was prepared. The same apparatus was also used for contact angle (CA) measurements using the sessile drop method. The polymer solution in methanol (0.3 mg/mL) was spin-coated on a thin glass plate. Next, deionized water (4 μL) was dropped onto the thin polymer layer, and the CA was measured. The data were collected and processed by SCA20_U software. The hydrodynamic diameters (D_h_) of particles and polydispersity indexes (PDI) were measured by dynamic light scattering (DLS) using a Zetasizer Nano-S90 (Malvern Technologies, Malvern, UK). Samples were placed in poly(methyl methacrylate) (PMMA) cells after dilution with a solvent (0.5 mg/mL). Then, they were put into the thermostatted cell compartment of the instrument at 25 °C. Each measurement was repeated three times to obtain an average value. Ultraviolet-visible light spectroscopy (UV-Vis, spectrometer Evolution 300, Thermo Fisher Scientific, Waltham, MA, USA) was used to determine the anionic drug content (DC) in conjugates or the non-ionic drug loading content (DLC) in micelles, as well as the amount of the drug released during in vitro studies. The measurements were carried out in quartz cuvettes.

## 3. Results

The graft copolymers were obtained by atom-transfer radical polymerization (ATRP) catalyzed with CuCl/bpy complex in THF/methanol at 40 °C. The backbone was constructed from copolymers of methyl methacrylate and 2-(2-bromoisobutyryloxy)ethyl methacrylate (P(MMA-*co*-BIEM)) with various contents of bromoester initiating groups (25% or 50%). Side chains, which were grafted from these active groups in the multifunctional macroinitiator (MI), represented the structure of copolymers of [2-(methacryloyloxy)ethyl]-trimethylammonium chloride, in different ratios, with methyl methacrylate (P(TMAMA-*co*-MMA)) (25:75; 50:50). The polymers contained various grafting degrees (DG = 26 or 46 mol.%), depending on the number of initiating groups (Table 1). The structure of graft copolymers was confirmed using the ^1^H NMR spectroscopy (Figure S1).

**Figure 2:**
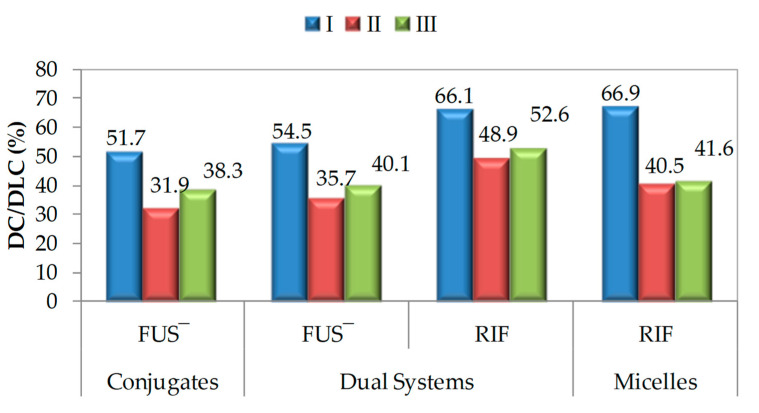
DC/DLC values of FUS or RIF for various carriers based on graft copolymers I–III, where: DC refers to the amount of conjugated ionic drug, and DLC relates to the amount of non-ionic drug encapsulated in the micelle core.

Graft copolymers I–III were used as the matrices to obtain different types of carriers (Figure 1). Chloride anions included in TMAMA units, which were distributed along the side chains in the polymer, served as ion-exchange species. Fusidate (FUS) sodium salt was selected as the API to obtain drug-carrier ionic conjugates. The efficiency of the exchange reaction using FUS anions was evaluated by the drug content (DC), which refers to the percentage of ionic drugs in the copolymer, determined by UV-Vis (Figure 2). The most effective exchange yielding DC > 50% took place in polymer I, which was characterized by a shorter main chain and loosely-distributed grafts (DG = 26 mol.%). A higher steric hindrance in the densely-grafted side chains (DG = 46 mol.%) likely caused tighter packing of the IL units, which corresponded to the lower efficacy of the Cl^−^ exchange to FUS^−^ in copolymers II–III (~35%).

The amphiphilic properties of graft copolymers I–III and their conjugates with FUS were determined by the critical micelle concentration (CMC). For this purpose, the interfacial tension (IFT) was measured using goniometry for the polymer/conjugate series in an aqueous solution, with a selected concentration range of *C* = 6 × 10^−4^–0.06 mg/mL. The crossover point on an IFT vs. log*C* plot was used to set the value of the CMC (Figure S2). The results in Table 2 show that the exchange of Cl^−^ to FUS^−^ changed the CMC for copolymer I (0.013 vs. 0.025 mg/mL), whereas these values were similar for copolymers with a higher graft density. The CMC values for FUS conjugates increased with the TMAMA fraction. The self-assembly behavior of the graft copolymers, including those bearing fusidate counterions, makes them suitable candidates for the encapsulation of non-ionic drugs to obtain dual-drug systems based on micellar conjugates.

**Figure 3:**
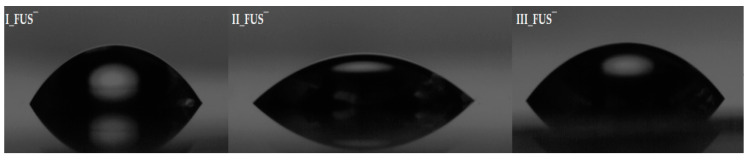
Screen shots during sessile water drop measurements for FUS conjugates.

Goniometry is a convenient method for measuring the water contact angle (CA, Table 2) using the sessile drop technique. The evaluation of a polymer film’s wettability indirectly describes the hydrophilic-hydrophobic balance in the macromolecule. It also helps show the specific influence of the polymer structure, including the type of counterion (Cl^−^ vs. FUS^−^). Polymer I, with the highest amount of hydrophilic TMAMA units in the grafts and the lowest molecular weight (I:F_TMAMA_ = 43%; M_n_ = 273.1 × 10^3^ g/mol), was characterized by the highest CA (56.3° and 51.0°, for carriers bearing Cl and FUS anions, respectively). The CA values slightly decreased after counterion exchange to FUS^−^ due to the higher hydrophilicity of the FUS^−^ systems. The differences in the water contact angles for the FUS conjugates are shown in Figure 3.

The amphiphilicity of the given structures allowed them to form micelles via self-organization. Therefore, both the copolymer with chloride counterions and conjugates with ionic drugs were used as the matrices for encapsulating the non-ionic drug rifampicin (RIF) in the micelle core. Rifampicin is a bactericidal antibiotic used to treat respiratory diseases caused by, e.g., *Mycobacterium tuberculosis*, *Streptococcus pyogenes*, and *Streptococcus pneumoniae*. The action mechanism of RIF is based on bacterial DNA polymerase blocking, which inhibits bacterial RNA and protein synthesis. Because of this, RIF is also combined with FUS to ensure an effective defense against many microorganisms that cause respiratory diseases. Marsot et al. also discovered drug–drug interaction between RIF and FUS [41]. Bel et al. noted that FUS increased the bioavailability and concentration of RIF in plasma, while decreasing its clearance [42]. Moreover, RIF can potentially induce the metabolism of FUS and reduce its concentration. Therefore, a dual-drug system formulation for the co-delivery of these agents (FUS and RIF) is clinically relevant. 

The degree of non-ionic drug encapsulation, namely the drug loading content (DLC), was calculated as a percentage of the drug loading concentration to the total concentration of the copolymer/conjugate and the loaded drug, using UV-Vis spectroscopy. Similar to FUS exchange, the encapsulation of RIF was the most efficient in copolymer I (Figure 2). It was also observed that the presence of an anionic drug in the polymer did not greatly impact its encapsulation (in micelles of a single-drug system, DLC_RIF_ = 66.9% vs. the dual-drug system, DLC_RIF_ = 66.1%). A similar trend was observed when II or III was used as the matrix, but they could encapsulate RIF in lower amounts, i.e., 50% and 40%, respectively. 

**Figure 4:**
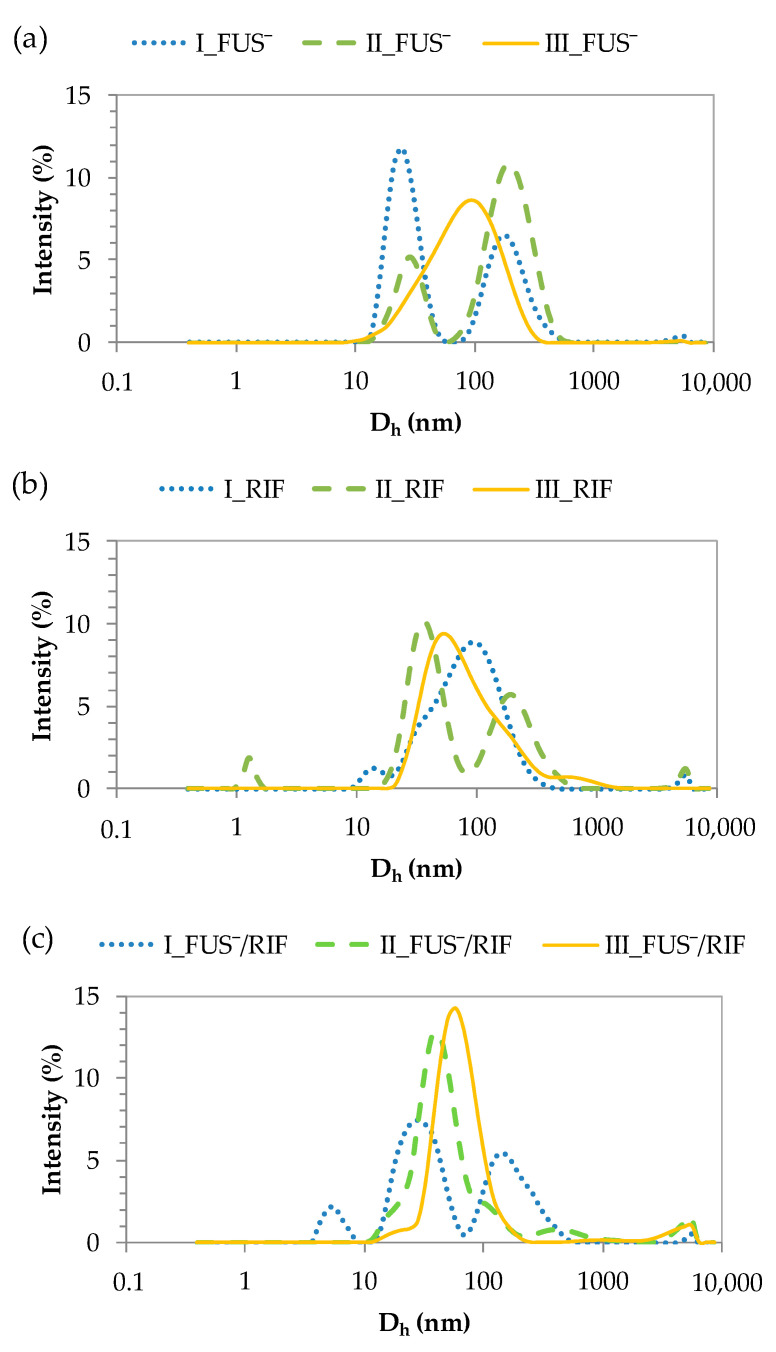
DLS histograms for particles in single systems with (**a**) FUS^−^, (**b**) RIF, and (**c**) dual systems.

The hydrodynamic diameters (D_h_) of the obtained carriers were determined by DLS in an aqueous solution. Figure 4 shows the histograms of polymer particles, and the detailed data are presented in Table S1. Compared with previously studied chloride-based copolymers [39], the introduction of FUS anions increased the particle sizes, except for system III (Cl^−^: 105 nm vs. FUS^−^: 95 nm). FUS^−^ conjugates I and II formed two fractions of particles at 30 nm and 200 nm. However, in the latter system, the larger particles were the dominant fraction. Similar differences were observed for both types of systems after the micellization of RIF, but the particle sizes of the dominant fraction were evaluated to be 30–40 nm (52–55%) and 51–97 nm (90–94%). The highest molecular weight of copolymer III helped generate monomodal particles, which depended on the system and reached sizes of 95 nm (FUS^−^ conjugate), 94 nm (RIF micelles), and 65 nm (micellar conjugate FUS^−^/RIF).

**Figure 5:**
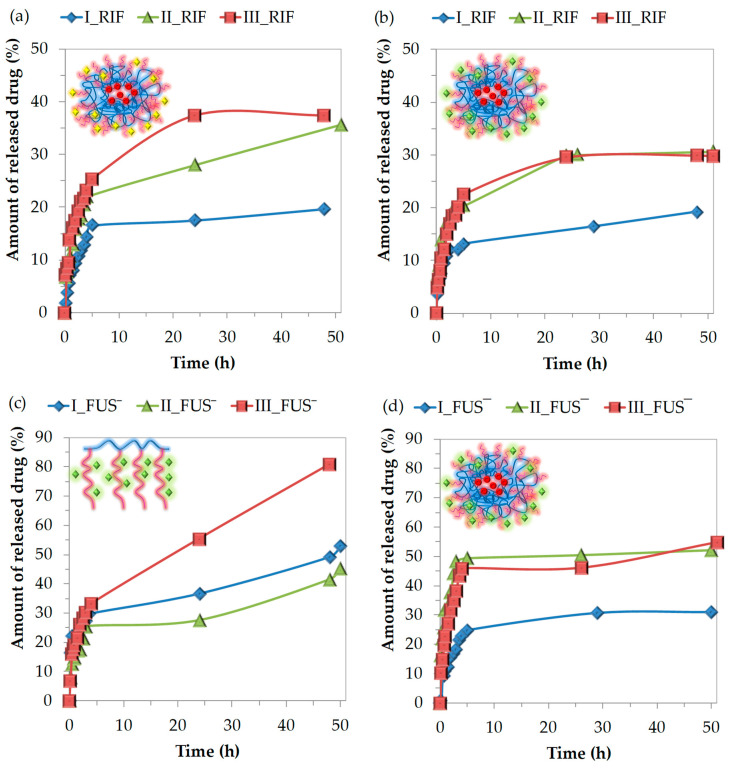
Kinetic release profiles of RIF from various types of carriers: (**a**) micelles and (**b**) dual-drug systems or FUS from (**c**) conjugates and (**d**) dual-drug systems, based on PIL graft copolymers I–III.

In vitro drug release studies were performed in PBS (pH = 7.4) for samples (0.5 mL) taken at appropriate time intervals and measured using UV-Vis at λ_FUS_ = 207 nm and λ_RIF_ = 330 nm. The release of ionic and/or non-ionic drugs (Figure 5) showed a biphasic kinetic dependence. An initial burst release was observed at up to 5 h, then a slower release lasted for up to 50 h. In the case of single-drug micelles I_RIF and III_RIF, as well as for all dual-drug systems, the drug release reached a plateau after 24 h. Generally, FUS was released in larger amounts from the conjugates than RIF was from the micelles, representing single-drug systems (Table 3). The highest difference in the release rate of RIF vs. FUS (micelles vs. conjugates), 37% vs. 80%, was observed for system III. This effect was reduced by using half-length grafts in system I (20% vs. 50%, respectively). Finally, the release rates were comparable with the shortest side chains, when DP did not exceed 30 units. The combination of FUS and RIF in the dual-drug system also caused a more rapid release of FUS anions than did the encapsulated RIF. Additionally, studies on the double systems indicated that the encapsulated RIF was released slightly more slowly than from the single-drug micelles. Similarly, the release of FUS anions was reduced compared with the single-drug conjugates. In all cases, the highest amount of the drug was released from the densely-grafted polymer III, which was characterized by the lowest fraction of TMAMA units (18%) in the longest side chains (DP_sc_ = 65), independent of the drug delivery form of the carrier. The final concentrations of co-released drugs indicated excess FUS, where the RIF:FUS ratio was equal to 0.7–0.8:1. Currently, pharmaceutical formulations containing both RIF and FUS are not commercially available, but Drancourt et al. proved the positive effect of this drug combination on drug-resistant strains in a ratio of 0.6:1 [43]. Therefore, our results for dual FUS/RIF systems are promising for the simultaneous delivery of two drugs, which could be applied in lung disease therapies against drug-resistant strains.

## 4. Conclusions

Graft copolymers with various contents of IL units were tested for obtaining three types of carriers, i.e., single-drug systems with conjugated FUS or encapsulated RIF, as well as dual-drug systems with conjugated FUS and encapsulated RIF. The drug delivery properties of these systems were verified. The ionic structure of the tested copolymers allowed for an ion exchange reaction of chloride anions to FUS^−^, which resulted in ionic drug-carrier conjugates. Both chloride-based copolymers and FUS conjugates showed the ability to self-organize; thus, they could be applied for the encapsulation of the non-ionic drug RIF in polymer micelle superstructures. The entrapment of RIF in the self-assembled conjugates with FUS^−^ was advantageous for achieving dual-drug systems for co-delivery applications. Drugs were successfully introduced into both conjugates and/or micellar carriers (FUS ≤ 54% and RIF ≤ 67%). There was no significant effect of the anion type (chloride vs. pharmaceutical fusidate) on the RIF encapsulation efficiency. The sizes of the self-assembled particles for the main fraction decreased in the following order: FUS conjugates (26–208 nm, 58–100%), RIF micelles (40–97 nm, 55–96%), and micellar conjugates FUS/RIF (31–65 nm, 52–95%). During in vitro studies in PBS, an initial burst release was observed. The amounts of the drugs released varied in a wide range, between 19–81%, depended on the side chain length and ionic content of the polymer, as well as the drug and carrier type. In conclusion, the selected trimethylammonium-containing graft copolymers are sufficient for obtaining single-drug systems in the form of micelles or ionic conjugates. They are also suitable as innovative dual-drug systems carrying two drugs, connected by a polymer matrix, in different ways (physically vs. ionically). The selected drugs (RIF and FUS) can be used for antibacterial treatment, including drug-resistant bacterial strains and combined therapy, with simultaneous drug co-delivery.

## Figures and Tables

**Table 1:** Data for P(MMA-*co*-(BIEM-*graft*-P(TMAMA-*co*-MMA))) graft copolymers synthesized by ATRP. Data from [39].

No.	n_sc_	DG (mol.%)	F_TMAMA_ ^a^ (mol.%)	DP_sc_^a^	M_n_ ^a^ × 10^−3^ (g/mol)	Ð ^b^
I	48	26	39	35	273.1	1.15
II	133	46	36	28	583.5	1.03 ^c^
III			18	65	1090.5	1.11

Conditions: I, II: [TMAMA]_0_:[MMA]_0_:[MI]_0_:[CuCl]_0_:[bpy]_0_ = 50:50:1:1:2, III: [TMAMA]_0_:[MMA]_0_:[MI]_0_:[CuCl]_0_:[bpy]_0_ = 25:75:1:1:2, methanol/THF = 2:1 *v*/*v*; 1:1 *v*/wt, 40 °C. The main chain MI_I_: MMA/BIEM = 75/25; DP_n_ = 186; MI_II-III_: MMA/BIEM = 50/50; DP_n_ = 292, where DP_n_ is the polymerization degree of the main chain. n_sc_ is the number of side chains; DG is the degree of grafting, equal to n_sc_ per total DP_n_ of the polymer backbone; DP_sc_ is the polymerization degree of the side chains; F_TMAMA_ is the content of TMAMA in the side chains; ^a^ determined with ^1^H NMR using monomer conversion calculated for the reaction mixture by estimating the integration of signals for unreacted TMAMA (6.07 ppm) and MMA (6.02 ppm) in relation to the constant intensity of the pyrene signal (8.26–8.18 ppm) as the internal standard; ^b^ determined by SEC (PEO calibration in DMF or ^c^ in H_2_O).

**Table 2:** Characterization of aqueous solution and surface wettability for graft copolymers and their conjugates with FUS^−^.

	CMC ^a^ (mg/mL)		CA ^b^ (°)	
	Cl^−^ (Data from [39])	FUS^−^	Cl^−^ (Data from [39])	FUS^−^
I	0.013	0.025	56.3	51.0
II	0.020	0.020	48.3	35.3
III	0.011	0.012	44.3	43.2

^a^ Evaluated using the crossover point of IFT and logC of polymer/conjugate; ^b^ estimated using the water sessile drop method on a polymer film by goniometry.

**Table 3:** Data for drugs released from carriers I–II based on TMAMA.

	Conjugates		Dual-Drug Systems				Micelles	
	FUS^−^		FUS^−^		RIF		RIF	
	ARD (%)	CD (μg/mL)	ARD (%)	CD (μg/mL)	ARD (%)	CD (μg/mL)	ARD (%)	CD (μg/mL)
I	52.82	7.18	30.84	4.31	19.19	3.29	19.65	3.37
II	45.23	3.80	52.11	4.65	30.57	3.88	35.64	3.70
III	81.32	8.21	54.84	5.57	29.91	4.03	37.37	3.98

ARD is the amount of released drug; CD is the concentration of the drug released after 48–50 h.

## Data Availability

Not applicable.

## Supplementary Materials

The following supporting information can be downloaded at: https://www.mdpi.com/article/10.3390/ma15134457/s1, Figure S1: Representative ^1^H NMR spectrum of graft copolymer I; Figure S2: Representative plot of interfacial tension vs. logarithm of the conjugate concentration II_FUS in aqueous solution at 25 °C; Table S1: Hydrodynamic diameters (D_h_) of nanoparticles determined using DLS.
