# Supplementary material for: Dual-Drug Delivery via the Self-Assembled Conjugates of Choline-Functionalized Graft Copolymers
Source: Materials (Basel). 2022 Jun 24;15(13):4457. doi: 10.3390/ma15134457 (PMC9267481; doi:10.3390/ma15134457)
Supplement: Supplementary file 1 [file materials-15-04457-s001.zip › materials-1768143-supplementary.pdf]

Supporting Information

Dual drug delivery via self-assembled conjugates of choline functionalized graft copolymers

Katarzyna Niesyto, Aleksy Mazur and Dorota Neugebauer *

* Department of Physical Chemistry and Technology of Polymers, Faculty of Chemistry, Silesian University of Technology, 44-100 Gliwice, Poland

* Correspondence: Dorota.Neugebauer@polsl.pl

Contents:

Figure S1. Representative ^{1}H NMR spectrum of graft copolymer I.

Figure S2. Representative plot of interfacial tension vs logarithm of the conjugate concentration II_FUS in aqueous solution at 25 ºC.

Table S1. Hydrodynamic diameters (D_h) of nanoparticles determined using DLS.

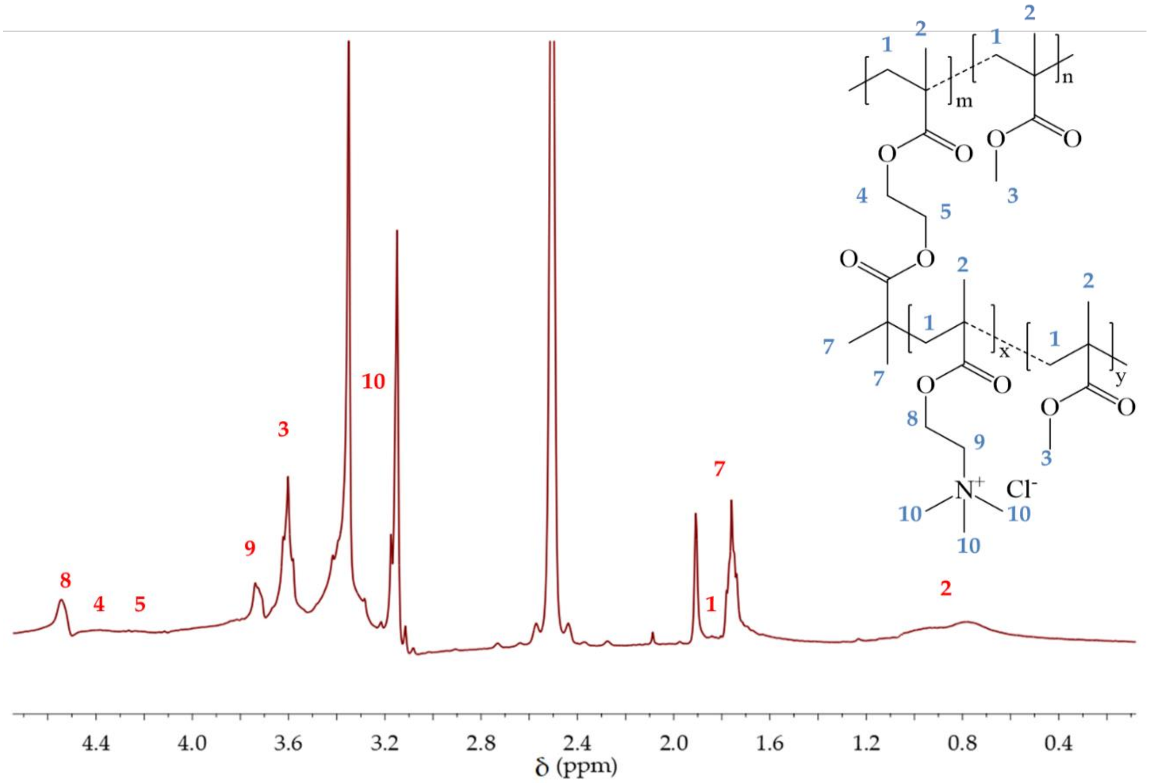

Figure S1. Representative ^{1}H NMR spectrum of graft copolymer I.

^{1}H NMR (DMSO-d$_6$, δ, ppm): 4.63-4.43 (2H, -C**H$_2$**-O-), 4.47-4.28 (2H, -C**H$_2$**-OOC-C-(CH$_3$)$_2$Br), 4.28-4.08 (2H, -COO-C**H$_2$**), 3.86-3.65 (2H, -C**H$_2$**-N$^+$), 3.65-3.47 (3H, -O-C**H$_3$**), 3.42-3.01 (9H, -N$^+$-(C**H$_3$**)$_3$, 1.98-1.82 (6H, -(C**H$_3$)$_2$**Br initiating moiety), 1.4-0.51 (3H, -C**H$_3$** backbone).

After exchange of Cl$^-$ by FUS$^-$, the new signals appeared in ^{1}H NMR (DMSO-d$_6$, δ, ppm): 0.7-0.9 (3x3H, -C**H$_3$** at ring, #14,15,21), 0.9-1.2 (3x2H and 3x1H, -C**H$_2$** in ring, #1(1H),2(2H), 6(2H),7(2H),10(1H),12(1H)), 1.25 (3H, -C**H$_3$** at ring, #22), 1.3-1.4 (2x1H, C**H** in ring, C**H**-CH$_3$, #8,4), 1.6-1.7 (2x3H, -C**H$_3$**, #19,20), 1.8 (3H, -OCOC**H$_3$** #24), 2.02-2.17 (2x2H, -C**H$_2$**, #16,17), 2.17-2.33 (3x1H, -C**H$_2$** in ring, #1(1H),10(1H),12(1H)), 4.0-4.2 (2x1H, C**H**-OH, #3,9), 5.10 (1H, -C**H**= #18), 5.88 (1H, -C**H**-COOCH$_3$ #13).

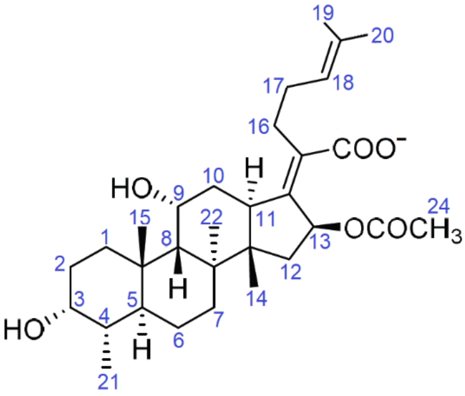

FUS$^-$

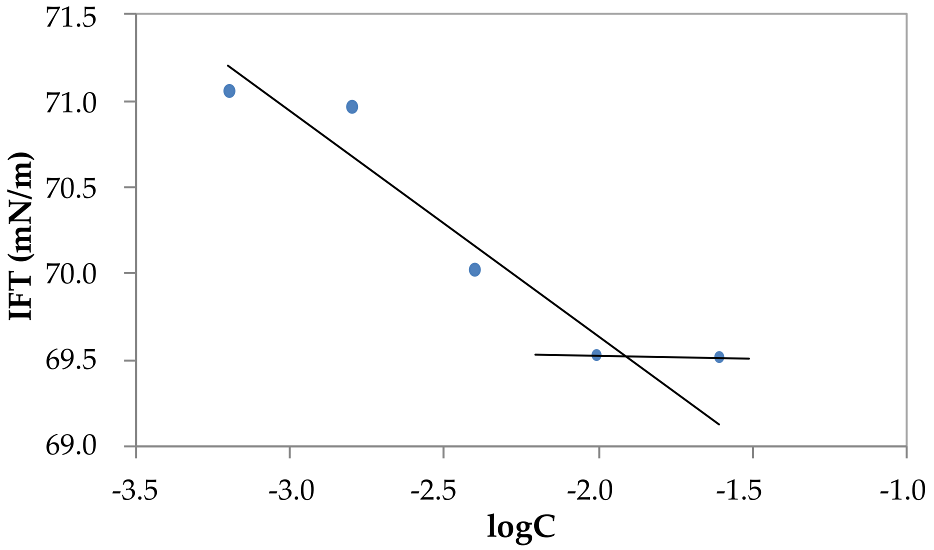

Figure S2. Representative plot of interfacial tension vs logarithm of the conjugate concentration II_FUS in aqueous solution at 25 °C.

Table S1. Hydrodynamic diameters (D_h) of nanoparticles determined using DLS[a].

	Cl^- (37)			FUS^-			RIF			FUS^-/RIF		
	PDI	Size (nm)	Intensity (%)	PDI	Size (nm)	Intensity (%)	PDI	Size (nm)	Intensity (%)	PDI	Size (nm)	Intensity (%)
I	0.454	18<br>125	64<br>32	0.424	26<br>199	58<br>41	0.436	97<br>14	94<br>5	0.23	31<br>184	52<br>40
II	0.241	72	100	0.564	208<br>29	83<br>7	0.377	40<br>216	55<br>40	0.371	51<br>531	90<br>6
III	0.293	105	99	0.281	95	100	0.269	94	96	0.27	65	95

[a]concentration of copolymer in water: 1 or 0.5 mg/mL.
